# Gastroenterological surgery in Japan: The past, the present and the future

**DOI:** 10.1002/ags3.12008

**Published:** 2017-04-25

**Authors:** Hugh Colvin, Tsunekazu Mizushima, Hidetoshi Eguchi, Shuji Takiguchi, Yuichiro Doki, Masaki Mori

**Affiliations:** ^1^ Department of Gastroenterological Surgery Osaka University Graduate School of Medicine Osaka Japan

**Keywords:** advances, gastroenterological surgery, history, Japan, progress

## Abstract

In the last two centuries, there has been remarkable progress in the field of gastroenterological surgery, including the curative resection of cancers, replacement of failed organs through transplantation, increased safety of undergoing major surgeries and decreased operative morbidity through developments in minimal access surgery. Japan has very much been at the forefront of these advances, as is evident from the present review, from advancing the surgical management of gastric cancer to the pioneering work in live‐donor transplantation. This review also highlights many instances where surgical management of the same pathologies has evolved differently between Japan and the West. It is encouraging that many procedures established in Japan are eventually taken up by the West, often after rigorous assessment affirming the quality and applicability of such techniques. In Japan, many of the crucial issues in gastroenterological surgery are increasingly addressed through large multi‐institutional prospective control trials, ensuring that Japanese surgeons continue to contribute to the advances in gastroenterological surgery.

## 1 Introduction

In 1804, Hanaoka Seishū began carrying out surgery under general anesthesia; he is widely acknowledged for being the first to do so, well before the first ether anesthetic was given by Crawford Long in 1842. This was a remarkable achievement considering that Seishū developed the general anesthetic himself, based on his knowledge of Chinese herbal medicine. He combined this with the European surgical techniques he had learned through apprenticeships, which were introduced to Japan at the time by the Dutch. Although the first operation he carried out was the removal of breast cancer, he went onto carry out many other procedures including treatment of hemorrhoids and fistula‐in‐ano.^1^

What Seishū achieved reflects the way in which the field of gastroenterological surgery has developed in Japan since his time; Japanese gastroenterological surgeons have been consistent in learning the very best practices of the time from home and abroad. In turn, Japanese surgeons have refined what they have learned, and have developed novel and better ways of managing diseases, thereby being very much at the forefront of the advances in gastroenterological surgery on the global stage.

It is an honor to be able to highlight some of the most notable achievements made by Japanese surgeons in the modern era, that have also had a global impact in the field of gastroenterological surgery. We apologize for omitting much of the significant and groundbreaking work as a result of the constraints of the length of this article. We are at the same time very proud of the achievements made by our colleagues of the past and the present. In analyzing the achievements to this date, we will also reflect on how we can move forward in the future to continue making advances in gastroenterological surgery.

## 2 Esophageal surgery

The first successful esophagectomy for carcinoma of the thoracic portion of the esophagus was conducted by Torek in 1913,^2^ but the procedure was associated with a very high mortality rate for many decades thereafter. In the middle of the 20th century, Nakayama achieved a strikingly low operative mortality rate of 16.7% (five of 30 individuals) in patients undergoing esophagectomy for cancer,^3^ aided by the use of endotracheal anesthesia and perioperative intermittent positive pressure ventilation. This was the lowest ever mortality rate to be reported at the time. The operative mortality rate fell further, partly as a result of Nakayama promoting the undertaking of esophagectomy and reconstruction over different time points, thereby limiting the operative stress.^4^

In Japan, since the early 1980s, a large proportion of esophageal cancers have been managed by three‐field lymph node dissection,^5^ based on data suggesting that the rate of isolated cervical lymph node metastasis could be as much as 40% in patients with squamous cell carcinoma.^6^ Large nationwide observational studies of patients who have undergone three‐field lymph node dissection have since confirmed that approximately one‐third of the patients harbor metastasis in the cervical nodes. Also, the overall long‐term survival was better in those who underwent three‐field compared to two‐field lymph node dissection, with comparable and low perioperative mortality rates.^7^, ^8^ The benefits of carrying out three‐field lymph node dissection have been viewed largely with scepticism in the West, because of the high rate of morbidity associated with this procedure, as well as the perception that the cervical lymph nodes are less likely to be involved in adenocarcinomas, which occur more commonly in the West and in the lower esophagus. However, a recent report by Altorki *et al*.^9^ from a large prospective observational study showed that approximately one‐third of patients with esophageal cancer had otherwise unsuspected cervical node involvement regardless of the histological type, and that three‐field lymph node dissection was associated with better long‐term overall survival. Therefore, in the future, three‐field lymph node dissection may become more frequent for treating esophageal cancer outside of Japan.

Although three‐field lymph node dissection has led to improvements in long‐term survival, surgery alone is associated with a limited 5‐year survival of approximately 40%, and adjuvant therapy plays an important role in improving the long‐term survival further. In the West, neoadjuvant chemoradiotherapy has become the standard treatment after being shown to be superior to chemotherapy alone when carrying out two‐field lymph node dissection for adenocarcinoma.^10^ The role of adjuvant therapy for squamous cell carcinoma of the esophagus for patients who receive three‐field lymph node dissection has been explored in several multi‐institutional randomized control trials in Japan. Survival benefit with adjuvant chemotherapy for esophageal cancer was first demonstrated, and a subsequent trial went on to show that neoadjuvant chemotherapy was superior to adjuvant chemotherapy in terms of overall survival, and the former has now become the standard treatment in Japan.^11^ The role of radiotherapy in addition to chemotherapy in the context of three‐field lymph node dissection for squamous carcinoma of the esophagus is still a matter of debate, and is being addressed in an ongoing nationwide randomized control trial by the Japan Clinical Oncology Group.^12^

## 3 Gastric surgery

Japanese surgeons have arguably had the most influence worldwide on the way gastric cancer surgery is carried out. The practice of gastrectomy with extended lymphadenectomy for gastric cancer was established in Japan and provides the foundation for effective treatment of this disease.^13^ In 1989, Maruyama *et al*.^14^ published the largest and most detailed work concerning lymph node metastasis in gastric cancer, and then later demonstrated the effectiveness of extended lymphadenectomy for gastric cancer.^13^ In the West, the higher prevalence of proximal gastric cancers, and patient factors such as older age, higher body mass index and higher incidence of comorbidities make the surgical management more challenging,^15^, ^16^, ^17^ has perhaps resulted in the tradition of carrying out a more limited lymph node dissection. Despite this, there is now a wider uptake of extended lymphadenectomy in countries other than Japan, and this is thought to be behind the significant improvement in long‐term survival.^18^, ^19^, ^20^, ^21^, ^22^

More recently, in Japan, a number of well‐powered multi‐institutional studies have evaluated the feasibility and the benefits of going beyond the field of dissection of D2 lymphadenectomy. Sasako *et al*.^23^ demonstrated that there was no benefit in terms of long‐term survival when using the left thoracoabdominal approach for gastric cancers of the cardia and subcardia (which allows better access to the mediastinal nodes) compared to the abdominal‐transhiatal approach, but that the former was associated with increased morbidity. In a separate trial, Sasako *et al*. was able to demonstrate the feasibility of carrying out para‐aortic dissection on top of D2 lymphadenectomy for gastric cancer, although there was no benefit to the 5‐year overall survival.^24^, ^25^ Most recently, Sano *et al*.^26^ showed that splenectomy for patients with proximal gastric cancer that does not involve the greater curvature was associated with increased morbidity without improving the overall 5‐year survival. These studies have thus been informative in defining the extent of dissection that should take place for gastric cancer.

Japanese surgeons have also sought ways to minimize the morbidity and mortality associated with carrying out gastrectomy. In 1994, Kitano *et al*.^27^ reported the first laparoscopic Billroth I gastrectomy and since then, gastrectomy is increasingly being carried out laparoscopically and in some cases, robotically in Japan.^28^ Most importantly, the results of two ongoing randomized control trials comparing laparoscopic and open gastrectomy for gastric cancer are awaited.^29^ In order to eliminate the occurrence of dumping syndrome post‐gastrectomy, Kodama *et al*.^30^ developed pylorus‐preserving gastrectomy for the treatment of gastric cancer, based on the technique pioneered by Japanese surgeons for the treatment of gastric ulcers.^31^ In 2000, Sasako *et al*.^32^ documented what is considered to be the gold standard for the management of complications after gastrectomy, leading to significant reductions in postoperative mortality in patients who suffer postoperative complications.

In terms of the management of advanced gastric cancers, there are also several notable achievements. Japanese surgeons have been involved with showing the benefits of S‐1 adjuvant chemotherapy for patients with locally advanced gastric cancer who have undergone D2 lymph node dissection.^33^ This was followed by the demonstration of cisplatin being beneficial on top of S‐1 as first‐line treatment for advanced and unresectable gastric cancer.^34^ Fujitani *et al*.^35^ were the first to properly address the issue for performing gastrectomy on top of chemotherapy in patients with incurable gastric cancer through a high quality randomized control trial. They conclusively showed that there was no benefit of performing gastrectomy in this context.

## 4 Hepatic surgery

Japanese surgeons have had significant involvement during the 20th century in achieving the drastic improvements in the safety and effectiveness (long‐term survival) of surgery for hepatocellular carcinoma. In 1949, Honjo *et al*.^36^ carried out the first successful anatomical right hepatectomy for hepatocellular carcinoma. Prior to this, partial hepatectomy and non‐anatomical resections were common practice. Liver resection was further refined by Takasaki *et al*.^37^ who pioneered the Gliossoneal pedicle approach at the hepatic hilus to carry out hepatectomy, allowing surgeons to undertake sectionectomies and segmentectomies in the cirrhotic liver. Accurate anatomical dissection of the liver requires a bloodless field, such as through the occlusion of the entire hepatic inflow through the Pringle maneuver. However, the use of such a maneuver must be balanced with the negative impact of ischemia on the remnant liver. In order to reduce the hypoxic stress to the liver during the Pringle maneuver, Makuuchi *et al*.^38^ developed hemihepatic vascular occlusion during liver resection.

Accurate intraoperative assessment of hepatocellular carcinoma and the hepatic anatomy through intraoperative ultrasound allows for accurate planning of hepatectomy, and this technique was popularized by Japanese surgeons during the 1980s. One of the significant advancements leading to the widespread use of intraoperative ultrasound was the development of an ultrasound probe designed to allow for direct contact with the liver surface by Makuuchi *et al*.,^39^ who subsequently went on to describe how the technique should be incorporated when carrying out subsegmentectomies.^40^

Preoperative portal venous embolization was pioneered by Makuuchi *et al*.,^41^, ^42^ who carried out the first case for hilar bile duct carcinoma to allow the remnant lobe of the liver to hypertrophy, thus allowing for extensive hepatectomy to take place without the occurrence of postoperative liver failure. Since then, this approach has been used to enable partial hepatectomy where the function of the remnant liver is diminished.

Given that cadaver transplantation is very rare in Japan, living‐donor liver transplantation has developed in our country. Makuuchi and his colleagues carried out the first living‐related adult to adult partial liver transplantation in 1993 for primary biliary cirrhosis and, since then, the indications have extended to the treatment of hepatocellular carcinoma.^43^ This has allowed a wider pool of patients to be treated, especially in Asian countries where the number of deceased organ donors is low.

## 5 Biliary surgery

In the 1950s, Kasai and Suzuki^44^ pioneered hepatoportoenterostomy for the treatment of congenital biliary atresia, which is now widely known as the ‘Kasai procedure’. This is now the first‐line treatment for relieving jaundice and preventing liver failure in those with biliary atresia.

In a single‐center, 34‐year review of 574 consecutive patients, Nagino *et al*.^45^ published the results of what is the world's largest cohort of patients who have undergone surgical treatment for perihilar cholangiocarcinoma. From their data, they demonstrated increasing use of major hepatectomy, and a reduction in perioperative mortality to 1.4%. Patients with R0 and N0 disease had a 5‐year disease‐specific survival of 67.1%, demonstrating in all that high‐quality surgery can offer a cure even for patients suffering from perihilar cholangiocarcinoma.

## 6 Pancreatic surgery

In 1960, Imanaga *et al*.^46^ described methods for reconstruction when carrying out pancreaticoduodenectomy based on studies they had done in animals and humans. Recommendations from this article include making a mucosa‐to‐mucosa anastomosis between the pancreatic duct and the jejunum, which led to better preservation of the exocrine pancreatic function, and is still used to this day.

Living‐donor pancreatic islet allotransplantation was pioneered by Matsumoto *et al*.^47^ in 2005. They successfully harvested islets from a donor who underwent distal pancreatectomy for the treatment of brittle diabetes mellitus in the recipient. The recipient subsequently became insulin independent, while the donor remained healthy with good glucose tolerance.

## 7 Colorectal surgery

In colorectal cancer surgery, some of the great achievements include the first successful one‐stage abdominosacral resection for rectal cancer by Ito *et al* in 1902^48^, who also noted the importance of the dissection of the upper rectal lymph nodes, prior to Miles carrying out abdominoperineal resection in 1908.^49^ Management of low rectal carcinoma has evolved differently in Japan from the rest of the world, such as those with stage II/III disease occurring below the peritoneal reflection commonly being managed by mesorectal excision and lateral lymph node dissection. Very recently, a large randomized control trial confirmed the superiority of carrying out lateral lymph node dissection together with mesorectal excision, this being associated with less local recurrence in patients with stage II/III rectal cancer below the peritoneal reflection.^50^

In 1995, Shirouzu *et al*.^51^ conducted a comprehensive pathological analysis of 610 specimens of rectal cancer that had been excised, and reported that a distant resection margin of 1 cm was sufficient in most patients, thus expanding the utility of sphincter‐preserving surgery in patients with rectal cancer.^52^ Closely following the publication of the restorative proctocolectomy with S pouch by Parks and Nicholls^53^ in 1978 for ulcerative colitis, in 1980, Utsunomiya *et al*.^54^ published the alternative J pouch.

It must also be mentioned that oxaliplatin, a platinum‐based antineoplastic agent used for colorectal cancer was discovered by Kidani *et al*.^55^ in 1976. As we know, oxaliplatin is now widely used as part of adjuvant chemotherapy, usually with folinic acid and 5‐fluorouracil, forming an important part of colorectal cancer management.

## 8 Endoscopic treatment of gastrointestinal cancers

For the treatment of early gastric cancer, endoscopic mucosal resection (EMR) is now well established in Japan. Data from Ono *et al*.^56^ included 479 gastric cancers treated by EMR and showed that 69% of these could be resected with a clear margin. Most importantly, the cancers without a clear resection margin could go on to be surgically resected or followed up endoscopically without any cancer‐related deaths, thereby showing that this less invasive approach could be carried out without compromising the cure rate. The latest method of endoscopic resection is endoscopic submucosal dissection (ESD), which allows for en bloc excision of early gastrointestinal malignancies, whether in the stomach, esophagus or large intestine. ESD enables a more accurate assessment of the depth of tumor invasion, allowing for more accurate prediction of the risk of lymph node metastasis. Cancer recurrence rates are very low after ESD, which has become an established practice for the treatment of early gastric and esophageal cancers in Japan, and is increasingly becoming used for early colorectal cancer.^57^ ESD is slowly becoming established in the West, suggesting that it will eventually become widely used worldwide.

## 9 Concluding remarks

Japanese surgeons have contributed significantly to the advancements in the field of gastroenterological surgery, including the way in which lymph node dissection is highly systematized and if necessary extensive for the management of gastroenterological malignancies. It is very encouraging that important surgical issues are increasingly being addressed through well‐designed randomized control trials. The present article illustrates many instances where surgical management of the same pathologies has evolved differently between Japan and the West, thereby making it even more important that Japanese surgeons continue to evaluate their practice rigorously and share their data with the rest of the world.

## Conflicts of interest

Authors declare no conflicts of interest for this article.
